# A Metastasis-Related lncRNA Signature Correlates With the Prognosis in Clear Cell Renal Cell Carcinoma

**DOI:** 10.3389/fonc.2021.692535

**Published:** 2021-06-03

**Authors:** Qian Dou, Shun Gao, Hua Gan, Zhao Kang, Han Zhang, Yichun Yang, Hang Tong

**Affiliations:** ^1^ Department of Nephrology, The First Affiliated Hospital of Chongqing Medical University, Chongqing, China; ^2^ Department of Urology, Mianyang Central Hospital, Mianyang, China; ^3^ Department of Oncology, Mianyang Fulin Hospital, Mianyang, China; ^4^ Department of Gastroenterology, The First Affiliated Hospital of Chongqing Medical University, Chongqing, China; ^5^ Department of Urology, The First Affiliated Hospital of Chongqing Medical University, Chongqing, China

**Keywords:** clear cell renal cell carcinoma, metastasis, lncRNA, prognostic signature, EMT

## Abstract

To explore the role of metastasis-related long noncoding RNA (lncRNA) signature for predicting the prognosis of clear cell renal cell carcinoma (ccRCC) patients. Firstly, metastasis-associated genes were identified to establish a metastasis-related lncRNA signature by statistical analysis. Secondly, the ccRCC patients were grouped into high-risk or low-risk group according to the established signature, and the different pathways between the 2 groups were identified by gene set enrichment analysis (GSEA). Finally, investigations involving PCR, transwell migration and invasion assay were carried out to further confirm our findings. The metastasis-related lncRNA signature was successfully constructed according to 7-metastasis-related genes (ADAM12, CD44, IL6, TFPI2, TGF-β1, THBS2, TIMP3). The diagnostic efficacy and the clinically predictive capacity of the signature were evaluated. Most of the values of the area under the time‐dependent receiver‐operating characteristic (ROC) were greater than 0.70. The nomogram constructed by integrating clinical data and risk scores confirmed that the risk score calculated from our signature was a good prognosis predictor. GSEA analysis showed that some tumor-related pathways were enriched in the high-risk group, while metabolism-related pathways were enriched in the low-risk group. In carcinoma tissues, the SSR3-6, WISP1-2 were highly expressed, but the expression of UBAC2-6 was low there. Knocking down SSR3-6 decreased the ability of migration and invasion in ccRCC cells. In conclusion, we successfully constructed a metastasis-related lncRNA signature, which could accurately predict the survival and prognosis of ccRCC patients.

## Introduction

As the most malignant urinary tumor in the mortality, the incidence of renal cell carcinoma accounts for 2% of adult malignant tumors, which is second only to prostate cancer and bladder cancer in urinary system (1). The ccRCC is the most common renal cell carcinoma, accounting for 80% of the cases. About 30% patients with ccRCC have already developed local or distant metastasis at the first visit, and the prognosis of these patients is often poor (2). Therefore, early diagnosis is an urgent task to improve the prognosis of ccRCC patients.

Epithelial-mesenchymal transition (EMT) is a progress that cancer cells transform from epithelial characteristics to acquire the mesenchymal cells characteristics. Regulated by multiple growth factors and transcription factors, EMT could destroy intercellular junction and contribute to tumor progression and metastasis (3). Although the EMT-related signatures have been found to be closely related to the metastasis and prognosis of ccRCC, there is still a lack of research on whether they could serve as biomarkers of early diagnosis and prognosis evaluation for ccRCC patients, which deserve deep study.

lncRNA is a kind of non-coding RNA that is longer than 200 nucleotides. lncRNA participates in many physiological and pathological processes, and the abnormal expression of lncRNA has been confirmed to be related to the proliferation, invasion, and migration of ccRCC cells, making it a potential biomarker to predict the survival outcomes of ccRCC patients (4, 5). In our study, we identified 7 metastasis-related genes markers through the comprehensive analysis of GEO database and the EMT-related genes. Moreover, a 7-metastasis-related lncRNA prognosis signature was successfully constructed, and the signature was verified and analyzed by combining the clinicopathological types.

## Materials and Methods

### Data Acquisition

The RNA-seq expression information were obtained from TCGA (https://portal.gdc.cancer.gov/), ICGC (https://dcc.icgc.org/) and GSE150404 from GEO database (https://www.ncbi.nlm.nih.gov). Clinical profiles of the ccRCC patients were obtained from TCGA and ICGC database (**Supplementary File S1**). 537 ccRCC samples from TCGA database (the TCGA whole set, n=537) were assigned into the training set (n=269) and the internal validation set (n=268) randomly using R software. The other 91 ccRCC samples from ICGC database were defined as the external validation set. The EMT-related genes (**Supplementary File S2**) were downloaded from The Molecular Signatures Database (https://broadinstitute.org/gsea/msigdb/).

### Identification of the 7 Metastasis-Related Genes

The GSE150404 dataset contained 15 ccRCC samples of Grade 1 (without metastasis) and 15 ccRCC samples of Grade 4 (with metastasis) in gene expression profiles. The identification of differentiation expressed genes (DEGs) (**Supplementary File S3**) was performed by GEO2R. P<0.05 and |logFC|>1.5 was set as the cut-off criteria. Biovenn (https://www.biovenn.nl/) was utilized to get the intersection of the DEGs and the 200 EMT-related genes, and finally 7 metastasis-related genes were identified.

### Identification of Metastasis-Related lncRNAs and Construction of Prognosis Signature

Pearson correlation analysis was conducted to identify the metastasis-related lncRNAs (**Supplementary File S4**). The correlation was calculated according to the expression value between lncRNAs and 7 metastasis-related genes. |R|>0.4 and P<0.05 were set as the cut-off criteria. In the training set, there were 47 metastasis-associated lncRNAs identified by univariate Cox analysis since they were closely related to the overall survival (OS) of the ccRCC patients (**Supplementary File S5**). Subsequently, a 7 metastasis-associated lncRNA signature was constructed by multivariate Cox regression analysis as independent prognosis factors in patient survival.

### Evaluation and Verification of the Accuracy of Prognostic Signature

The metastasis-related lncRNA signature was constructed with training set and verified with the TCGA whole set, the internal and external validation set. The risk scores were calculated as following:

$$Score=\Sigma_{i=1}^{n}\;coef(i)\times x(i),$$

where *coef*(*i*) represented the regression coefficient and *x*(*i*) represented the expression of each metastasis-related lncRNA. The ccRCC patients were grouped into high-risk and low-risk group. ROC curve was constructed to evaluate the diagnostic efficacy of our signature. The efficiency of the risk score on predicting the survival were assessed by Cox regression analyses. Moreover, the stratified analysis of ccRCC patients was performed using the “survival” and “survminer” packages of R software.

### Establishment of the Nomogram

A nomogram was established based on age, gender, stage, and risk score. The nomogram was assessed by drawing the calibration curve and ROC curve.

### Gene Set Enrichment Analysis (GSEA)

The “c2.cp.kegg.v7.2.symbols.gmt” KEGG gene sets was obtained from the Molecular Signatures Database. The P−value was obtained after performing 1000 permutation, and the enriched gene sets were obtained when P<0.05.

### Patients and Tissue Specimens

The ccRCC tissues and normal control tissue were obtained from the ccRCC patients who underwent radical nephrectomy at the Department of Urology of the First Affiliated Hospital of Chongqing Medical University. This study was approved by the Human Research Ethics Committee of the First Affiliated Hospital of Chongqing Medical University.

### Cell Culture and Treatment

The ccRCC cell lines (786-O, RCC-JF, RCC-23, Caki-1) and human renal tubular epithelial cell HK-2 were obtained from the ATCC (Manassas, USA). The 786-O, RCC-JF and HK-2 were cultured in RPMI 1640 medium (Corning, USA) containing 10% fetal bovine serum (FBS) (Gibco, USA). RCC-23 was cultured in Dulbecco’s modified Eagle’s medium (DMEM) (Gibco, USA) containing 10% FBS. Caki-1 was cultured with McCoy’s 5A medium (Biological Industries, Israel) containing 10% FBS. All cells were kept in 5% CO_2_ incubator at 37°C. Cell transfection was performed with Lipofectamine 2000 (Invitrogen, USA) followed the manufacturer’s protocol. 786-O was seeded into the six-well plates (8×10^4^cells/well). When the confluency of cells reached to 70%, exchanged the medium with 1.5 mL of basal medium containing 500 ul of Lipofectamine 2000 mix and siRNA.

Small interfering RNA:

si-SSR3-6-1#:
5′-CUGACAUGUUCUCAUUUAATTUUAAAUGAGAACAUGUCAGTT-3′.
si-SSR3-6-2#:
5′-CCUCAAAUAACUCACUUUATTUAAAGUGAGUUAUUUGAGGTT-3′.
si-SSR3-6-3#:
5′-CUCCCUUCUUCACUUACAATTUUGUAAGUGAAGAAGGGAGTT-3′.

### Quantitative Real-Time PCR

Total RNA was extracted with TRIzol^®^reagent (Takara Biotechnology Co., China), and the cDNA samples were synthesized using random primers and a Reverse Transcriptase PCR kit (Takara Biotechnology Co, China). The expressions of lncRNA SSR3-6, WISP1-2, CYP4F22-3 and UBAC2-6 were quantified by real-time PCR. GAPDH was used as the internal control.

The SSR3-6 primers:

forward: 5′-TCCCTCTACCACCCCATAGC-3′.
reverse: 5′-ATGCTTTTCCCCAGTGCTACC-3′.
WISP1-2,
forward: 5′-CTGAGACCTCTTGCCTGACG-3′.
reverse: 5′-TGCAGTGAACACCTTGACCT-3′.
CYP4F22-3,
forward: 5′-AAGGACTCCGCCGAAGAAT-3′.
reverse: 5′-AACAGTTGATCCTCCCACCAG-3′.
UBAC2-6,
forward: 5′-CAGAGGTTTCATAGCCGCCA-3′.
reverse: 5′-TTATTGAGTGCGGACGGCAT-3′.

### Cell Migration and Invasion Assays

Transwell migration assay was applied to detect the migration ability of 786-O cells. At 48h post-transfection, cells were collected and resuspended in serum-free RPMI 1640. Then cells (6×10^4^/200µL) were loaded into the upper chamber, and the lower chambers contained 600 μL of medium with 10% FBS. After 8 h incubation, the cells under the membrane were fixed with 4% paraformaldehyde and stained with 0.5% crystal violet. Except for that a transwell chamber with Matrigel was used instead, the experimental steps of invasion assay were performed almost the same as that of the migration assay.

### Statistical Analysis

Data were presented as the mean ± standard deviation. GraphPad Prism 8 software (GraphPad Software, CA) and R software were used for statistical analyses. Analysis of variance or t test was used for statistical comparisons. *p<0.05 was considered statistically significant.

## Results

### Identification of the 7 Metastasis-Related Genes

**Figure 1:**
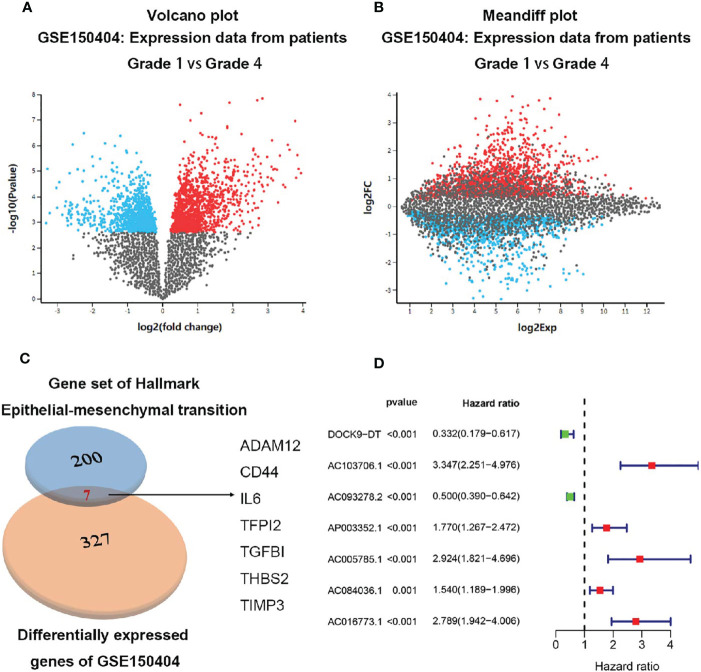
Identification of the 7 metastasis-related genes. **(A)** Volcano plot of expression data from patients grade 1 vs grade 4 in GSE150404. **(B)** Meandiff plot of expression data from patients grade 1 vs grade 4 in GSE150404. **(C)** Biovenn was utilized to get the intersection of the 327 DEGs and the 200 EMT-related genes, and finally 7 metastasis-related genes consisting of ADAM12, CD44, IL6, TFPI2, TGF-β1, THBS2, TIMP3 were identified. **(D)** 7 metastasis-related lncRNAs were supposed to be independent prognostic factors in ccRCC patients after multivariate Cox regression analysis.

A total of 214 up-regulated DEGs and 113 down-regulated DEGs were identified from GSE150404 (cut off criteria: p<0.05 and |logFC|>1.5) (**Figures 1A, B**). By analyzing the intersection of the 327 DEGs and the 200 EMT-related genes, finally 7 metastasis-related genes (ADAM12, CD44, IL6, TFPI2, TGF-β1, THBS2, TIMP3) were identified (**Figure 1C**).

### Construction of the Signature of Metastasis-Related lncRNA in Patients With ccRCC

**Figure 2:**
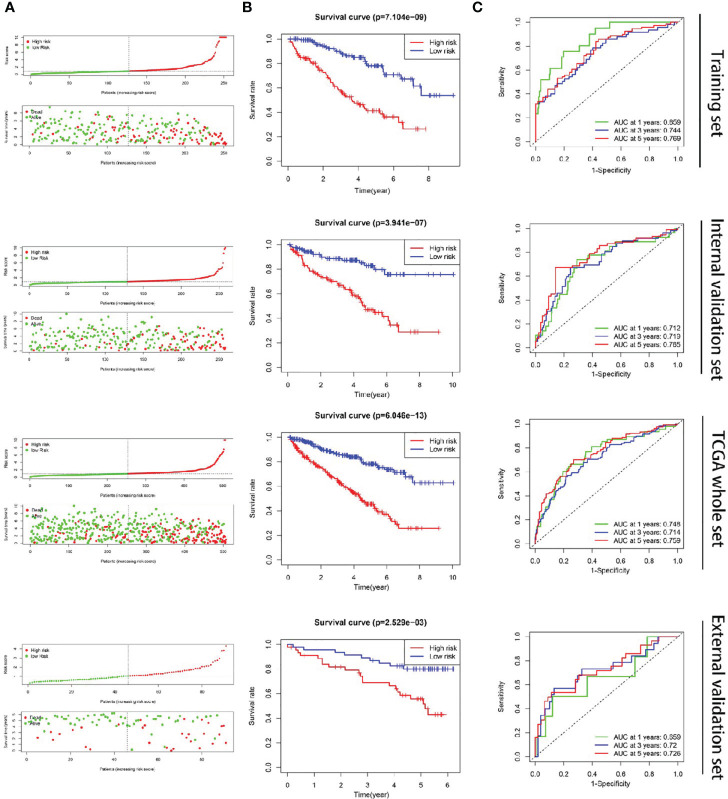
Construction of the metastasis-related lncRNA signature in ccRCC. **(A)** The ccRCC patients were divided into two groups according to the risk score. **(B)** The OS period was longer in the low-risk group than that of the high-risk group. **(C)** The diagnostic efficacy of the metastasis-related lncRNA signature was evaluated by the time-dependent ROC curves, in which all the AUC were more than 0.70 except for the “1 year” of external validation set.

192 metastasis-related lncRNAs were identified through Pearson correlation analysis of the lncRNAs from the ccRCC cases and the 7 metastasis-related genes. In training set, 47 lncRNAs were identified to be associated with the OS of ccRCC patients after univariable Cox regression analysis between the 192 metastasis-related lncRNAs and the survival data (**Supplementary File S5**). Moreover, 7 metastasis-related lncRNAs were supposed to be independent prognostic factors in ccRCC patients after multivariate Cox regression analysis between the 47 lncRNAs and the survival data (**Figure 1D** and **Table 1**). The ccRCC patients were grouped according to the risk scores (**Figure 2A**), and the low-risk group had a longer OS period (**Figure 2B**). The diagnostic efficacy of the metastasis-related lncRNA signature was evaluated by the ROC curves, in which all the area under the ROC (AUC) were more than 0.70 except for the “1 year” in external validation set (**Figure 2C**). These results above suggested that the metastasis-related lncRNA signature could effectively predict survival period in ccRCC patients.

**Table 1:** 7 metastasis-related lncRNAs significantly associated with the OS of ccRCC patients.

Gene symbol	Aliases	Ensemble ID	Location	pvalue	HR
DOCK9-DT	UBAC2-6	ENSG00000260992	chr13:99,086,723-99,090,425	0.000479	0.33225
AC103706.1	WISP1-2	ENSG00000261220	chr8:133,572,171-133,573,861	2.36E-09	3.347276
AP003352.1	HSALNG0067185	ENSG00000245970	chr8:98,041,726-98,044,121	0.000817	1.769677
AC005785.1	CYP4F22-3	ENSG00000268189	chr19:15,379,076-15,381,194	8.96E-06	2.924243
AC093278.2	ZNF366-1	ENSG00000261269	chr5:72,439,899-72,442,387	5.16E-08	0.500479
AC084036.1	SSR3-6	ENSG00000272990	chr3:156,523,740-156,524,247	0.001091	1.54036
AC016773.1	RF00019	ENSG00000207009	chr4:1,683,420-1,683,529	2.81E-08	2.789384

### The Metastasis-Related lncRNA Signature Was Associated With ccRCC Progression

**Figure 3:**
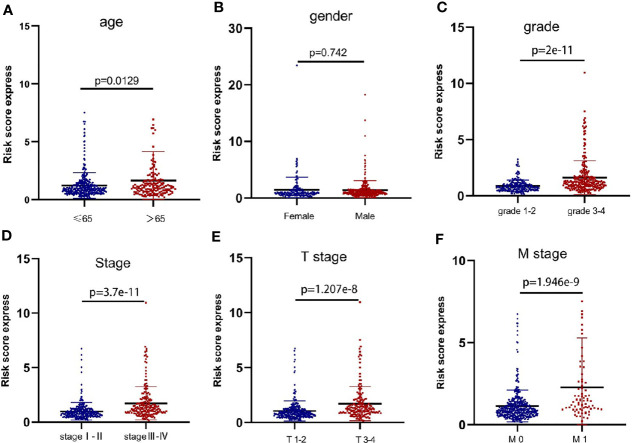
The metastasis-related lncRNA signature was associated with ccRCC progression. The correlation between the signature risk scores and the clinicopathological features, such as **(A)** age (<=65 vs >65, p=0.0129), **(B)** gender (Female vs Male, p=0.742), **(C)** grade (grade 1-2 vs grade 3-4, p=2e-11), **(D)** Stage (stage I-II vs stage III-IV, p=3.7e-11), **(E)** T stage (T stage 1-2 vs T stage 3-4, p=1.207e-8), **(F)** M stage (M stage 0 vs M stage 1, p=1.946e-9).

We found no significant correlation between the risk scores and gender of ccRCC patients (p=0.742) (**Figure 3B**). However, patients in>65 years, Grade 3-4, Stage III-IV, T stage 3-4, and M stage 1 groups showed significantly higher risk scores compared with patients in <=65 years (p=0.0129) (**Figure 3A**), Grade 3-4 (p<0.001) (**Figure 3C**), Stage I-II (p<0.001) (**Figure 3D**), T stage 1-2 (p<0.001) (**Figure 3E**), and M stage 0 (p<0.001) (**Figure 3F**) groups, separately. These results suggested that the metastasis-related lncRNA signature was associated with ccRCC progression.

### The Metastasis-Related lncRNA Signature Was an Independent Factor

**Figure 4:**
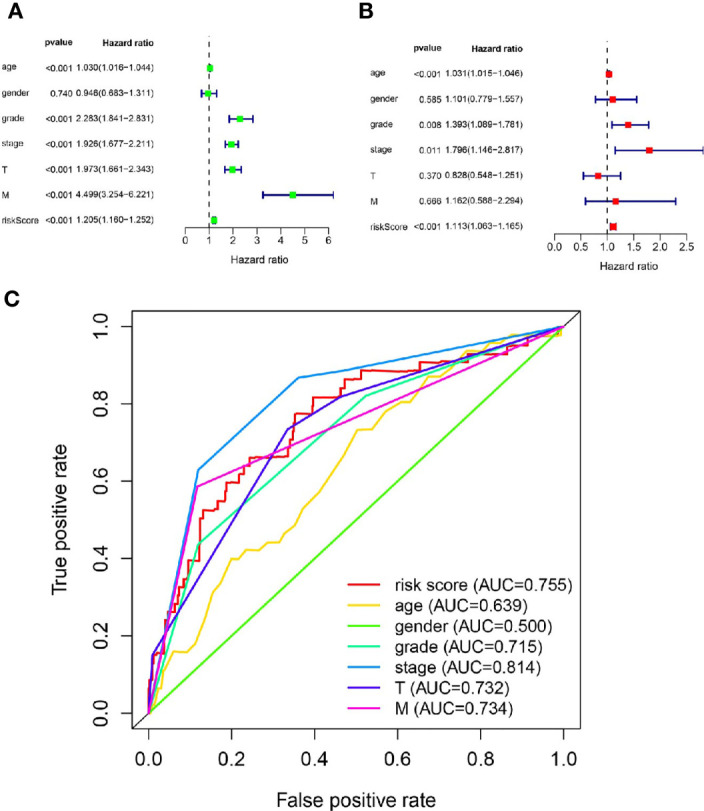
The metastasis-related lncRNA signature was an independent factor. **(A)** The correlation between OS and age, gender, grade, stage, T stage, M stage, risk score was performed by Univariate Cox regression analysis. **(B)** The correlation between OS and age, gender, grade, stage, T stage, M stage, risk score was performed by Multivariate Cox regression analysis. **(C)** The AUC of the ROC curve showed the prognostic accuracy of age, gender, grade, stage, T stage, M stage and risk score.

The results of Cox regression analysis showed that the risk score calculated from our signature was significantly related to the OS of the ccRCC patients (**Figures 4A, B**). The ROC curve analysis showed that the AUC value of the risk score was 0.755, which was the second highest (**Figure 4C**).

### Stratification Analyses

**Figure 5:**
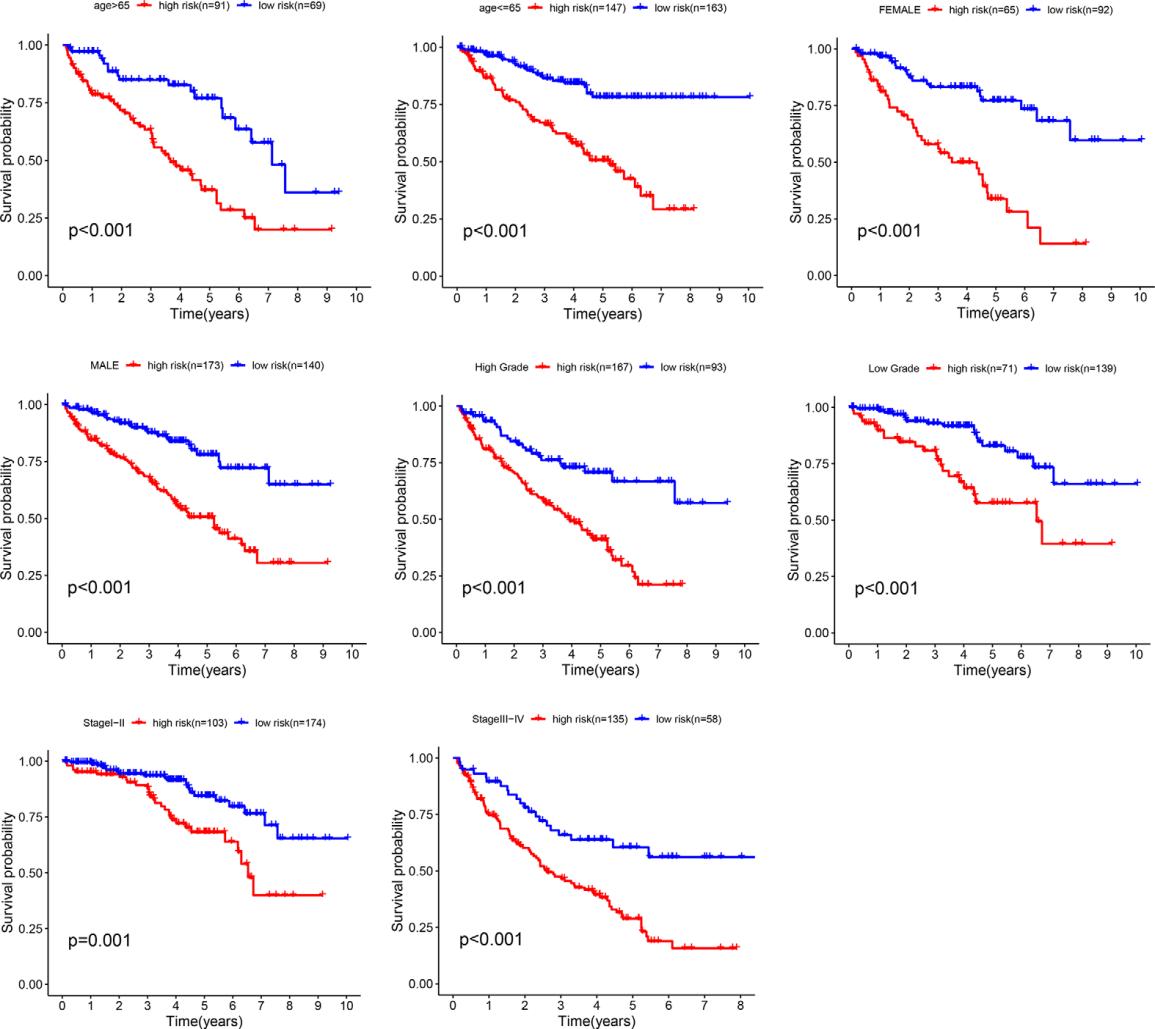
Stratification analyses. Survival curve analysis showed the OS rates of the high-risk and low-risk groups stratified by age, gender, grade and stage.

The stratified analysis based on clinicopathological information (**Figure 5**) showed that the ccRCC patients in the high-risk group had shorter OS period in different stratums, such as age (>65 years or <=65 years), gender (male or female), Grade (Grade 3-4 or Grade 1-2), Stage (Stage III-IV or Stage I-II). The result suggested that our metastasis-related lncRNA signature was powerful to predict the survival period of ccRCC patients in different gradation of age, gender, Grade and Stage.

### Establishment of the Nomogram

**Figure 6:**
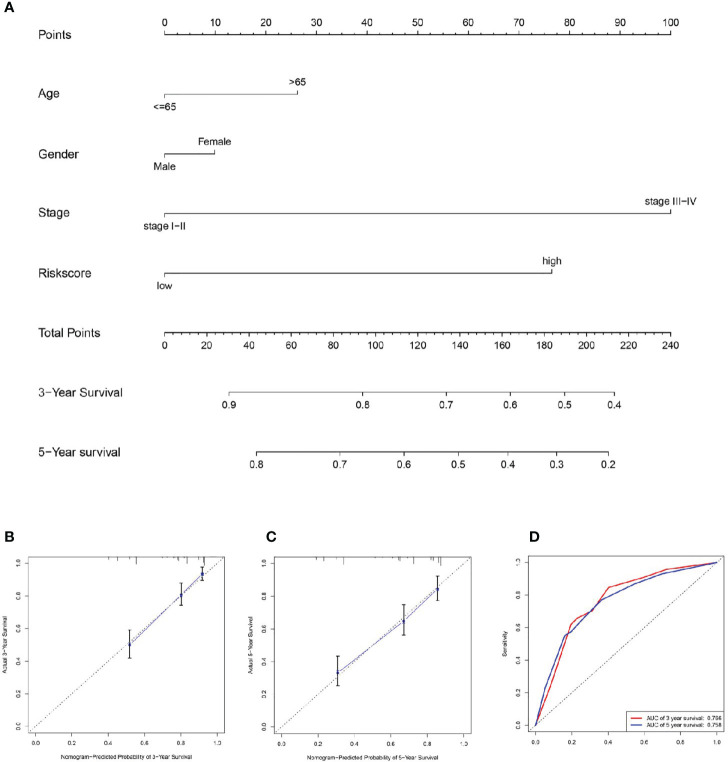
Establishment of the nomogram. **(A)** We plotted a nomogram based on age, gender, stage, and signature risk score. By drawing the calibration curve **(B, C)** and ROC curve **(D)** to assess the nomogram.

A nomogram was plotted based on age, gender, stage, and signature risk score (**Figure 6A**). The calibration plots predicted the 3-year and 5-year OS more accurately than the reference line did (**Figures 6B, C**). The AUC of the nomogram in the ROC curves were 0.766 and 0.758 at 3-year and 5-year respectively (**Figure 6D**).

### GSEA

**Figure 7:**
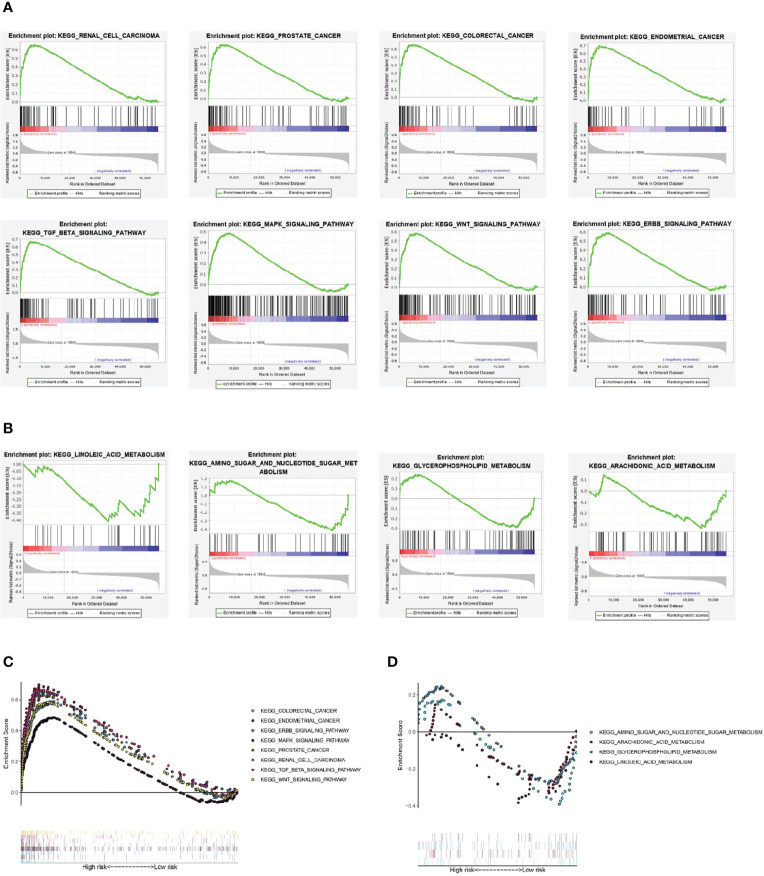
Gene set enrichment analysis. **(A, C)** Renal cell carcinoma, colorectal cancer, endometrial cancer, prostate cancer, ERBB signaling pathway, MAPK signaling pathway, WNT signaling pathway, and TGF-β signaling pathway were significantly enriched in the high-risk group. **(B, D)** amino sugar and nucleotide sugar metabolism, arachidonic acid metabolism, glycerophospholipid metabolism, linoleic acid metabolism were significantly enriched in the low-risk group.

The results of GSEA showed that some cancer and tumor progression-related pathways (renal cell carcinoma, colorectal cancer, endometrial cancer, prostate cancer, ERBB signaling pathway, MAPK signaling pathway, WNT signaling pathway, and TGF-β signaling pathway) were enriched significantly in the high-risk group (**Figures 7A, C**). While the metabolism-related signal pathway (amino sugar and nucleotide sugar metabolism, arachidonic acid metabolism, glycerophospholipid metabolism, linoleic acid metabolism) were significantly enriched in the low-risk group (**Figures 7B, D**).

### Expression of the Metastasis-Related lncRNAs in ccRCC

**Figure 8:**
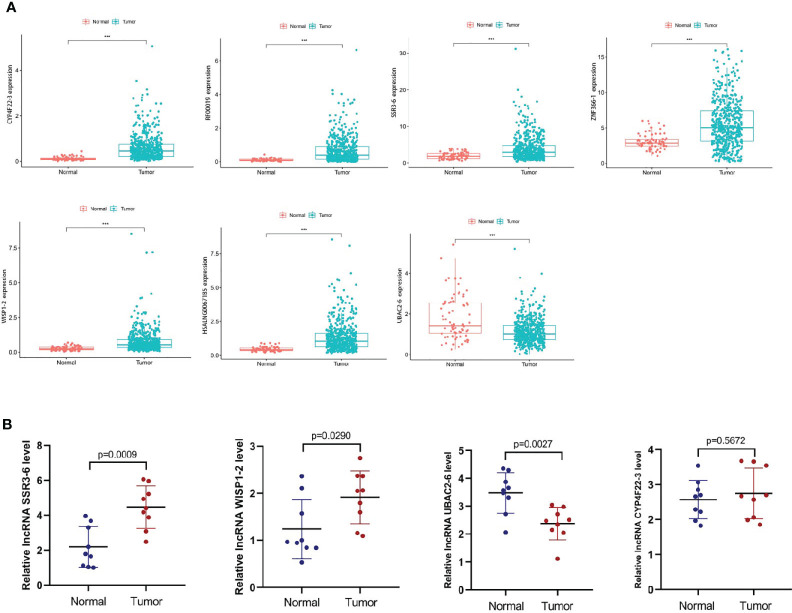
Expression levels of the metastasis-related lncRNAs in ccRCC. **(A)** We predicted the expression levels of the 7 metastasis-related lncRNAs in TCGA database (normal=72, tumor=539). **(B)** We detected the expression levels of SSR3-6, WISP1-2, CYP4F22-3 and UBAC2-6 in carcinoma and adjacent tissues of ccRCC patients (normal=9, tumor=9). SSR3-6, WISP1-2 was highly expressed in carcinoma tissues, UBAC2-6 was highly expressed in adjacent tissues.

We predicted 7 metastasis-related lncRNAs expression in TCGA database (normal=72, tumor=539) (**Figure 8A**). According to the correlation between the predictive expression of 7 metastasis-related lncRNAs (**Figure 8A**) and the OS of ccRCC patients from TCGA database (**Supplementary File S6**), the expression of SSR3-6, WISP1-2, CYP4F22-3 and UBAC2-6 were identified in carcinoma and adjacent tissues of ccRCC patients (normal=9, tumor=9) (**Figure 8B**). Among them, SSR3-6, WISP1-2 was highly expressed in carcinoma tissues, and UBAC2-6 was highly expressed in adjacent tissues.

### Knockdown of SSR3-6 Inhibits Cell Migration and Invasion of ccRCC Cells

**Figure 9:**
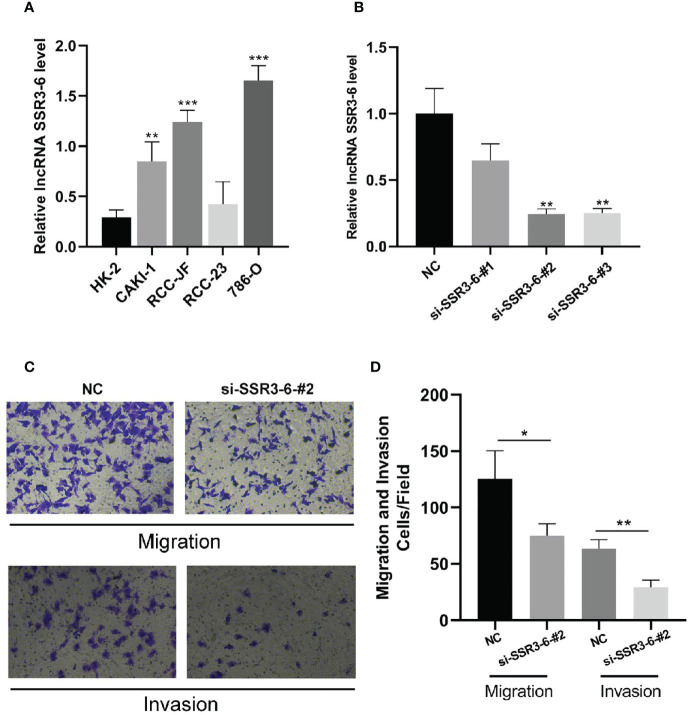
Knockdown of SSR3-6 inhibits cell migration and invasion of 786-O cells. **(A)** We detected the expression of SSR3-6 in HK-2 cells and 4 kinds ccRCC cells. SSR3-6 expression levels were higher in all ccRCC cells than in HK-2, and 786-O was the highest. **(B)** We used siRNA technology to knock down SSR3-6 in 786-O and found that the knockdown effect of si-SSR3-6-2# sequence was the best. **(C, D)** To investigate the biological role of SSR3-6 in migration and invasion, transwell assays were performed. We found that the cell migration and invasion abilities were markedly decreased in the si-SSR3-6-2#, compared with the NC groups. *P < 0.05, **P < 0.01, ***P < 0.001.

According to our experiments, the difference expression of SSR-3 in carcinoma and adjacent tissues was the most significant (**Figure 8B**), so the involvement in migration and invasion processes was investigated for SSR3-6. Compared with the HK-2 cells, the expression of SSR3-6 was higher in all ccRCC cells, in which the 786-O was the highest (**Figure 9A**). Next, we used siRNA technology to knock down SSR3-6 in 786-O and found that the knockdown effect of si-SSR3-6-2# sequence was the best (**Figure 9B**). The transwell assays showed that the ability of cell migration and invasion was markedly decreased after si-SSR3-6-2# knockdown (**Figures 9C, D**).

## Discussion

The ccRCC is the most common type of kidney cancers, accounting for 80% of all cases and over 30% of patients have metastases at the time of diagnosis (2). Molecular targeted drugs are the main drug therapy of metastatic renal cell carcinoma, which can prolong the OS and progression free survival (PFS) of the patients. However, the drug resistance and non-sufficient understanding of molecular markers of ccRCC metastasis have brought great difficulties to clinical treatment (6). Therefore, it is very important to find out sensitive and specific tumor markers and the mechanisms underlying the ccRCC invasion, metastasis and progression to improve the survival rate of ccRCC patients.

A number of studies have confirmed that EMT plays a key role in the whole process of tumor metastasis and colonization. At the beginning, the tumor cells located in the original site lose their epithelial cell characteristics, and the intercellular adhesion is no longer as tight as before. Then, the deepithelializated-characteristics tumor cells fall off from the basement membrane and get the characteristics of mesenchymal cells, so as to obtain a stronger ability of movement and invasion. With the circulation, these cells are taken to the metastasis destination, and the surviving tumor cells begin to adapt to the new tissue environment, and finally develop into secondary tumors scattered in the distal organs (7). Therefore, EMT-related markers are closely related to metastasis. In this study, we analyzed the gene expression in different Grade of ccRCC and found out DEGs in Grade 1 and Grade 4 in GEO database. Biovenn was utilized to get the intersection of the DEGs and the 200 EMT-related genes, and finally 7 metastasis-related genes consisting of ADAM12, CD44, IL6, TFPI2, TGF-β1, THBS2, TIMP3 were identified. ADAM12 belongs to the ADAMs family, which can promote cell invasion and metastasis in various cancers including esophageal cancer, colorectal cancer, breast cancer and prostate cancer (8–10). CD44 is a member of cell adhesion family and a transmembrane glycoprotein receptor encoded by a single gene. By binding to the ligand molecules, CD44 participates in the specific adhesion between cells and between cells and matrix. A large number of studies have shown that CD44 is related to tumor metastasis, invasion and prognosis (11–14). IL-6 is a multifunctional cytokine, which plays an important role in regulating immune response, hematopoietic system, tumor metastasis and endocrine system (15, 16). TFPI2 is a matrix related serine protease inhibitor with a Kunitz domain, and plays an important role in angiogenesis, tumorigenesis and metastasis (17, 18). As the most important factor in inducing EMT, TGF-β1 can promote the metastasis of various tumor cells (19). THBS2 is involved in many cellular biological processes by binding EMC protein and cell surface receptor (20). In cervical cancer, microRNA-221-3p promotes cervical cancer metastasis by directly targeting THBS2 (21). TIMP3 belongs to the TIMP family and acts as a dual regulator of the extracellular matrix remodeling and inflammation. Abnormal expression of TIMP3 has been observed in ccRCC (22). In brief, the 7 genes are closely related to tumor metastasis and the 7 genes-related biomarkers are potential prognosis and progression biomarkers for ccRCC patients.

lncRNA accounts for a large proportion (about 98%) in the genome of higher life. Compared with the protein coding sequences, lncRNA plays a more important role in regulating gene expression by affecting chromatin modification, RNA splicing and protein activity, thus affecting the occurrence, development, prognosis and chemotherapy resistance of tumors (4). More and more studies have found that lncRNA, as a new biomarker, may be a good predictor of ccRCC prognosis. It’s reported that a three-immune-related lncRNA signature could predict the survival of ccRCC patients (23), and a nine-redox-related lncRNA signature could serve as an efficient prognostic indicator for ccRCC (24). What’s more, a hypoxia-lncRNA assessment model may be useful to improve the prognostic prediction of ccRCC patients with the same tumor stage (25).

In this study, we constructed a signature of metastasis-related lncRNA in ccRCC patients, and the signature was verified to be associated with ccRCC progression which could be used as an independent predictor of the survival period. The nomogram (26) based on age, gender, stage, and signature risk score was also verified to be excellent in accuracy. We further analyzed GSEA pathway enrichment in high-risk and low-risk groups patients and found that the metabolism-related pathways were significantly enriched in the low-risk group while ERBB signaling pathway, MAPK signaling pathway, WNT signaling pathway, and TGF-β signaling pathway were significantly enriched in the high-risk group. ERBB is a tyrosine kinase type receptor. When ERBB binds to its ligand, it will be phosphorylated to activate the downstream signaling pathway, affecting the occurrence and progression of tumor (27). MAPK pathway is key signaling pathway that conducts extracellular signal through tertiary kinase cascade and regulates many physiological processes, such as cell growth, apoptosis and death. MAPK pathway is also of great significance in the malignant progression of tumor (28). TGF-β and Wnt signaling pathways are classical signaling pathways that activate EMT and promote tumor progression. WNT pathway can activate TGF-β pathway through TCF4, and TGF-β pathway can in turn activate WNT signal pathway through samd3 (29, 30). These pathways enriched in the high-risk group may be related to the metastasis of ccRCC.

Further experiments such as qRT-PCR, transwell migration/invasion assay were carried out to further confirm our findings. In our research, SSR3-6, WISP1-2 was highly expressed in carcinoma tissues, UBAC2-6 was highly expressed in adjacent tissues. What’s more, knocking down SSR3-6 decreased the migration and invasion ability of 786-O cells. These results suggested that the metastasis-related lncRNA participated in the invasion and migration of ccRCC cells. Our research also has some limitations. The specific mechanism of the effect of SSR3-6 on the invasion and metastasis of ccRCC cells remains to be further explored.

In conclusion, for the first time, we identified 7 metastasis-related genes through GEO database and The Molecular Signatures Database creatively. Next, according to the 7 metastasis-related genes, a metastasis-related lncRNA signature, which could accurately predict the survival and prognosis of ccRCC patients was constructed. The nomogram based on the risk score and clinical indicators of the signature had good predictive effect in predicting the prognosis of ccRCC patients. GSEA showed that tumor-related pathways were associated with high-risk group, and metabolism related pathway was associated with low-risk group. These results provided valuable insights for future research on the potential individualized treatment of ccRCC patients.

## Data Availability Statement

The original contributions presented in the study are included in the article/**Supplementary Material**. Further inquiries can be directed to the corresponding author.

## Ethics Statement

The studies involving human participants were reviewed and approved by Ethics Committee of the First Affiliated Hospital of Chongqing Medical University. The patients/participants provided their written informed consent to participate in this study.

## Author Contributions

HT and QD designed the research and revised the manuscript. HT and QD performed the experiments and wrote the draft manuscript. HT, QD, SG, HG, HZ, YY, and ZK analyzed the experimental results. HG and HZ wrote the Marked manuscript for review purposes. All authors contributed to the article and approved the submitted version.

## Conflict of Interest

The authors declare that the research was conducted in the absence of any commercial or financial relationships that could be construed as a potential conflict of interest.
