# Supplementary figures and images for: A Metastasis-Related lncRNA Signature Correlates With the Prognosis in Clear Cell Renal Cell Carcinoma
Source: Front Oncol. 2021 Jun 3;11:692535. doi: 10.3389/fonc.2021.692535 (PMC8209488; doi:10.3389/fonc.2021.692535)

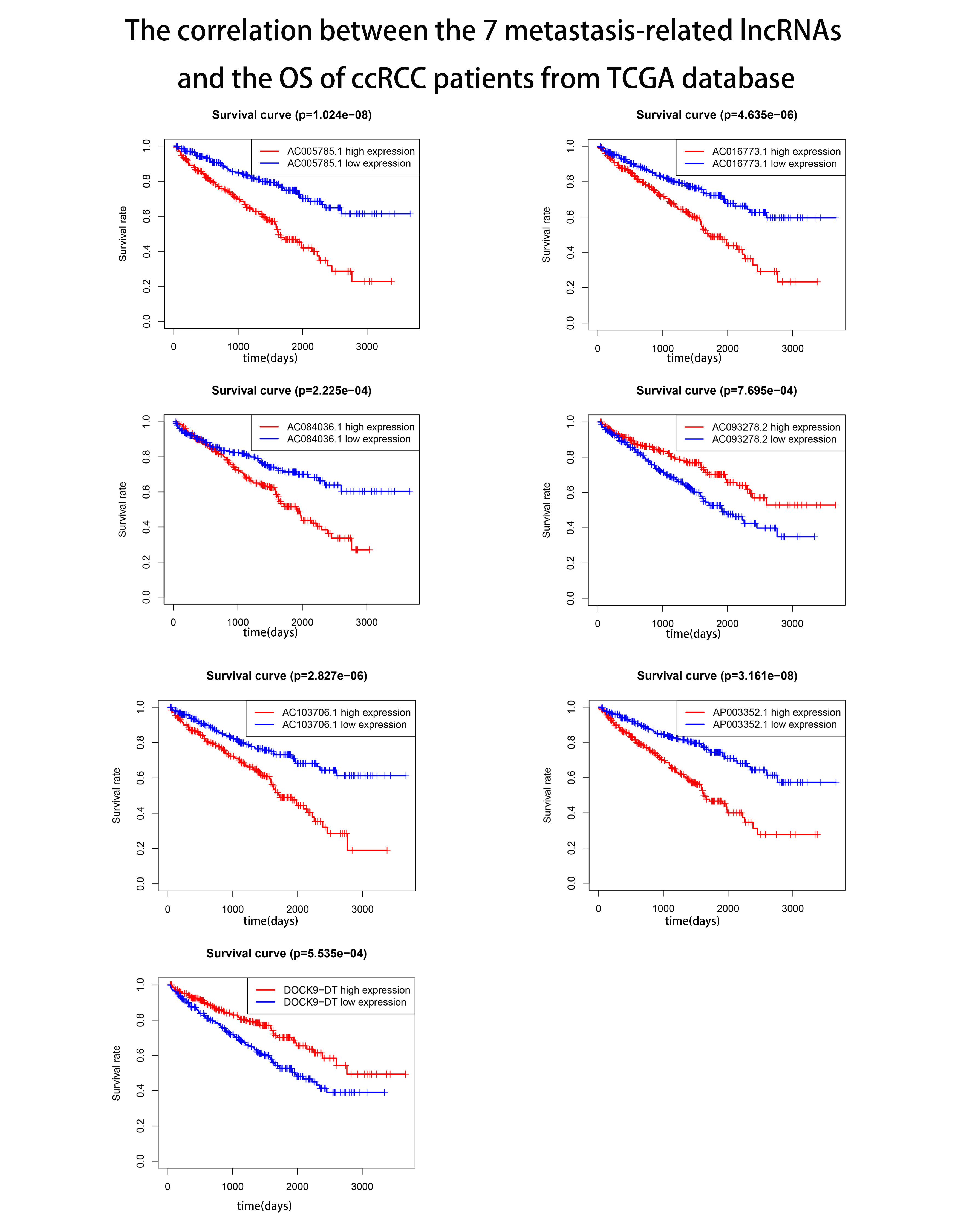

Supplement: Supplementary file 6 [file Image_1.tif]
